# Dr. Candler’s Book

**Published:** 1908-03

**Authors:** 


Dr. Candler's Book.—Everyday Diseases of Children and Their Rational Treatment. Geo. H. Chandler, M. D., Chicago. The Clinic Publishing Co., 1907. Cloth. 277 pages. $1.

The city physician has access to large libraries, and when in doubt or trouble he has the benefit of consultation with learned specialists. The everyday doctor, the village and country doctor, who must be an all-around specialist, is called on to practice every branch in the broad domain of medicine; he has no such resources. He must work out his problems alone; must think it out. He is expected to be "up" in every branch, and ready without warning or time for preparation to meet every emergency. To him, Dr. Candler's book will be a treasure trove, a very help in time of need. It embodies a large amount of Dr. Candler's experiences and observations under circumstances like the above mentioned, when, having to depend solely upon himself, he had to work out things by the bedside. It is a small volume and can be carried in the medicine case, buggy or saddle bags, and is arranged for ready reference. Children's diseases often puzzle the wisest. One has to learn to interpret baby language. They have a way of telling where and what hurts, a sign language; and you must learn it by observation. I will give an illustration: I was called about midnight to see a crying baby of six months who "just wouldn't go to sleep." Parents had done everything—examined for pins, suckled, rocked and bye-byed him, but to no purpose. I took him in my arm and he led me to the water bucket. Water was what he wanted, and he "wouldn't be happy till he got it." I gave him a gourd full and he fell asleep on my shoulder. Papa gave me $5 for going two miles to give baby a gourd of water.

Dr. Candler has had many such experiences, and his book is an epitome of his observations, his conclusions, his remedies used, and hygienic methods. It is chock full of really useful suggestions, diagnostic hints and therapeutic indications. He writes me: "I believe this little book has a mission, and have taken infinite pains to make it helpful; the kind of helpfulness I used to look for (and never found) during my early years of practice. If it should find favor in the eyes of the profession, I intend to enlarge the scope of the book, and hope, later, to make it a 'practice.'"

I confidently recommend this little work to my readers, and especially to the younger generation as really "worth while."

---

I CALL ATTENTION to Dr. Garrett's paper in this issue. Every doctor should read it and heed it. Few know the far-reaching and destructive effects of these diseases—diseases that have made gynecology a necessary branch of practice. It is an evil that cries aloud for reform, and emphasizes the necessity of a National Sanitary Code enforced by the Federal government. It is comprehended in the scope of the action contemplated and in motion by the Association for the Advancement of Science. See leading editorial.

---

THE NEW PRACTICE ACT is a veritable drag-net which will sift out many incompetents, some of whom, when first-course students, bought license from certain district boards. But it will also shut out some competent, popular and successful practitioners of years standing, who thoughtlessly failed to register their diploma as required by previous law. It is a maxim that ignorance of the law is no extenuation for its violation; and these gentlemen who failed to register, through indifference or ignorance, will have to be examined. Pretty hard on some of them.

---

DR. F. B. MOORE, of Palestine, died suddenly in that city, February 16th, ult.

